# Effects of host-associated low-temperature probiotics in olive flounder (*Paralichthys olivaceus*) aquaculture

**DOI:** 10.1038/s41598-024-52491-9

**Published:** 2024-01-25

**Authors:** Su-Jeong Lee, Da-In Noh, Young-Sun Lee, Md Tawheed Hasan, Sang Woo Hur, Seunghan Lee, Seong-Mok Jeong, Jong Min Lee, Eun-Woo Lee, Kang-Woong Kim, Won Je Jang

**Affiliations:** 1 Biopharmaceutical Engineering Major, Division of Applied Bioengineering, Dong-Eui University, Busan, 47340 South Korea; 2 Present Address: Department of Aquaculture, Sylhet Agricultural University, Sylhet, 3100 Bangladesh; 3 Department of Biotechnology, Pukyong National University, Busan, 48513 South Korea; 4 Aquafeed Research Center, National Institute of Fisheries Science, Pohang, 37517 South Korea; 5 Core-Facility Center for Tissue Regeneration, Dong-Eui University, Busan, 47340 South Korea

**Keywords:** Applied microbiology, Industrial microbiology, Biotechnology, Immunology, Microbiology

## Abstract

This study investigated the effects of supplementation of low-temperature probiotics isolated from the intestines of olive flounder on the growth performance, digestibility, and regulation of intestinal microbiota and the expression of genes related to growth, immunity, and apoptosis in olive flounder. Bacteria showing high growth at approximately 15–20 °C, which is the temperature of olive flounder culture, were isolated and confirmed to be *Pseudomonas* species through 16S rRNA gene sequence analysis. Whole-genome sequencing revealed that the strain has a 6,195,122 bp single circular chromosome and a guanine–cytosine content of 59.9%. In the feeding trial, supplementation with 1 × 10^8^ CFU/g of the isolate strain positively modulated growth performances, digestive enzyme activity, and gut microbiota composition of olive flounder. RT-qPCR for the comparison of growth, immunity, and apoptosis-related gene expression levels showed no significant differences between the groups. Therefore, the isolated host-associated low-temperature probiotics improved the growth performance of olive flounder by causing positive changes in digestive activity and intestinal microbial composition without affecting host gene expression.

## Introduction

In aquaculture, one of the most important essential nutrient for fish growth is a protein source; until 2005, the most cost-effective source of protein was fish meal^1^. However, as the production and supply of fishmeal become unstable because of a decrease in ocean fisheries stocks worldwide, the price of fishmeal is rising^1,2^. Consequently, fishmeal is no longer a cost-effective protein source; therefore, a new protein source that can yield a high protein content, provide a stable supply, and have a low price should be developed^3^.

Plant-derived protein sources such as soybean, corn gluten, and canola meal show potential as alternative protein sources because of their high protein content, large-scale production, and low price^4–8^. However, these plant-derived protein sources have low contents of some essential amino acids, such as lysine and methionine; they also have indigestible carbohydrates and anti-nutritional factors, which can interfere with nutrient digestion and absorption^8–10^. Therefore, animal protein sources with good digestibility and low carbohydrate content (such as poultry by-products and tankage meal) or additives that can increase digestibility (such as probiotics) can be used in appropriate combinations^3,11,12^.

Probiotics are defined as living microorganisms that provide beneficial health effects to the host when they are appropriately administered; as such, studies have been performed on the development of probiotics for humans as well as pets, livestock, and farmed fish^13–15^. In the early days, many studies targeted single lactic acid bacteria; recently, various approaches, including host-associated probiotics (HAPs), have been applied^16^. HAPs are bacteria isolated from the host’s gastrointestinal tract and may be more suitable for probiotic development than bacteria isolated from other sources as they are safe and well adapted within the host’s defense system^17^.

Olive flounder is commercially farmed in East Asian countries such as China, Japan, and Korea; since it is a carnivorous fish with high protein requirements, the feed used for flounder aquaculture contains a high fishmeal content^18,19^. The development of low-fishmeal feed is essential for sustainable flounder farming. In previous studies, alternative protein sources for flounder farming were investigated, and probiotics were developed to increase their digestibility^20^. However, most of the developed probiotics were isolated at 37 °C, or the optimum activation temperature was not investigated. Considering that the growth temperature of flounder is 20 °C, probiotics that grow well and have a high activity at this temperature should be developed for efficiency.

Therefore, this study investigated the characteristics of host-associated low-temperature probiotics (HALPs) isolated from wild flounder intestines and the effect of dietary supplements to develop HALPs suitable for the growth temperature of flounder.

## Materials and methods

### Bacterial isolation and identification

Bacteria were isolated from the intestine of wild olive flounder caught off the coast of Yeongdo (35°04′16.2″ N 129°05′08.0″ E). Briefly, the intestine was homogenized and suspended in phosphate-buffered saline (PBS). The suspension was serially diluted with PBS, poured into a brain heart infusion (BHI) agar plate, which is rich in nutrients and can cultivate various microorganisms, and incubated at 20 °C for 24 h. The cultured single colony was identified by performing 16S ribosomal RNA gene sequence analysis, mixed with 50% glycerol, and stored at − 80 °C until it was used.

### Bacterial whole genome sequencing

The whole-genome sequence (WGS) of the isolated bacteria was analyzed. The total DNA of the selected bacteria was extracted using the NucleoSpin Microbial DNA Mini kit (Macherey Nagel, Germany)^21^ and sequenced using an Oxford Nanopore MinION (NFEC-2022-08-281084) at the KNU NGS Center (Daegu, South Korea).

### Experimental diet preparation

The basal diet composition is shown in Table 1. The raw materials and composition ratios of the feed used were provided by the National Institute of Fisheries Science (Pohang, South Korea). FM70 with 70% fishmeal content and FM35 with 35% fishmeal content were prepared. A HALP-added feed was made by spraying on FM35, and the added probiotic concentration was 1 × 10^8^ CFU/g. It was sprayed and dried before feeding every day.

**Table 1:** Dietary formulation of the experimental diets for juvenile olive flounder.

Ingredients	Percent (%)	FM35	HALP
	FM70		
Fish meal (Chile)	35.00	17.50	17.50
Fish meal (Peru)	35.00	17.50	17.50
Tankage meal	–	11.50	11.50
Poultry by-product meal	–	6.50	6.50
Wheat gluten	–	4.70	4.70
Soy protein concentrate	–	8.00	8.00
Tuna by-product meal	–	1.00	1.00
Starch	4.00	3.75	3.75
Soybean meal	12.00	12.00	12.00
Wheat flour	7.00	7.00	7.00
Fish oil	3.30	4.20	4.20
Lecithin	0.50	0.70	0.70
Betaine	–	0.50	0.50
Taurine	–	0.50	0.50
Lysine	–	0.30	0.30
Methionine	–	0.25	0.25
Monocalcium phosphate	0.50	1.30	1.30
Mineral mix	1.00	1.00	1.00
Vitamin mix	1.00	1.00	1.00
Vitamin C	0.10	0.10	0.10
Vitamin E	0.10	0.10	0.10
Choline chloride	0.50	0.60	0.60
Probiotics (CFU/g)	–	–	1 × 10^8^

### Animals and experimental management

A total of 270 juvenile olive flounders with an average weight of 10.83 ± 0.26 g were obtained from a commercial hatchery (Jeogu Susan, South Korea). After 2 weeks of acclimatization, fish were randomly divided into 120 L semi-recirculating tanks (30 fish/tank, triplicates) and fed with the feed twice daily up to apparent satiation. The following aquatic environmental parameters were maintained throughout the experiment: temperature, 20.0 °C ± 0.5 °C; pH, 7.3 ± 0.3; salinity, 31 ± 1 ppt; and dissolved oxygen, 6.0 ± 0.5 mg/L. Seawater was provided after pre-filtration and UV sterilization.

### Growth performance, feed utilization, and body indices

Growth performance, feed utilization, and body indices were calculated by measuring fish weight, length, visceral weight, and liver weight after 24 h of fasting on the last day of week 8. These parameters were calculated as follows:

$${\text{Weight}}\;{\text{gain}}\;\left( {{\text{WG}},\;\% } \right) = \left( {{\text{final}}\;{\text{weight}} - {\text{initial}}\;{\text{weight}}} \right)/{\text{initial}}\;{\text{weight}} \times 100$$

$${\text{Specific}}\;{\text{growth}}\;{\text{rate}}\;\left( {{\text{SGR}}, \, \% /{\text{day}}} \right) = \left( {\ln \;{\text{final}}\;{\text{weight}} - \ln \;{\text{initial}}\;{\text{weight}}} \right)/{\text{days}} \times 100$$

$${\text{Feed}}\;{\text{conversion}}\;{\text{ratio}}\;\left( {{\text{FCR}}} \right) = \left( {{\text{final}}\;{\text{weight}} - {\text{initial}}\;{\text{weight}}} \right)/{\text{dry}}\;{\text{feed}}\;{\text{intake}}$$

$${\text{Protein}}\;{\text{efficiency}}\;{\text{ratio}}\;\left( {{\text{PER}}} \right) = \left( {{\text{final}}\;{\text{weight}} - {\text{initial}}\;{\text{weight}}} \right)/{\text{protein}}\;{\text{fed}}$$

$${\text{Condition}}\;{\text{factor}}\;\left( {{\text{CF}},\;\% } \right) = {\text{body}}\;{\text{weight}}/{\text{body}}\;{\text{length}}^{3} \times 100$$

$${\text{Viscerosomatic}}\;{\text{index}}\;\left( {{\text{VSI}},\;\% } \right) = {\text{visceral}}\;{\text{weight}}/{\text{body}}\;{\text{weight}} \times 100$$

$${\text{Hepatosomatic}}\;{\text{index}}\;\left( {{\text{HSI}},\;\% } \right) = {\text{liver}}\;{\text{weight}}/{\text{body}}\;{\text{weight}} \times 100$$

### Serum nonspecific immune and biochemical parameter analysis

Superoxide dismutase (SOD) activity was expressed as the percentage of superoxide inhibition by using a SOD activity colorimetric assay kit (BioVision, USA) in accordance with the manufacturer’s instructions. The respiratory burst (RB) generated by phagocytes was measured via a nitro blue tetrazolium assay (Sigma-Aldrich, USA) as previously described^22^. Myeloperoxidase (MPO) activity was analyzed with an MPO colorimetric activity assay kit (Sigma-Aldrich, USA). Antiprotease activity was expressed as the percentage of trypsin inhibition by using the method of Ellis^23^. Alanine aminotransferase (ALT), aspartate aminotransferase (AST), total glucose, total protein, triglyceride, and total cholesterols levels were measured with a BS-390 chemistry analyzer (Mindray Bio-Medical Electronics, China) at the Core-Facility Center for Tissue Regeneration, Dong-eui University (Busan, South Korea).

### Digestive enzyme activity

Intestinal digestive enzyme activity was analyzed in fish midgut samples and evaluated using amylase, lipase, and protease assay kits (BioVision, USA) in accordance with the manufacturer’s instructions.

### Intestinal microbiota analysis

Total bacterial DNA was extracted from the intestines of olive flounder randomly selected from each group by using the PureHelix™ genomic DNA prep kit (NanoHelix, South Korea). The quality of the extracted DNA was examined using a NanoDrop Lite spectrophotometer (Thermo Scientific, USA) and gel electrophoresis. A library was constructed in accordance with 16S Metagenomic Sequencing Library Preparation (Illumina, USA), and the sequence of the V3-V4 region was analyzed using the Illumina MiSeq System (Illumina, USA). Sequence data were analyzed with the Quantitative Insights into Microbial Ecology pipeline (http://qiime.org) via the SILVA database. The divisive amplicon denoising algorithm 2 pipeline was used to filter, denoise, merge, and remove chimeras of sequences. All samples generated rarefaction curves to confirm the tendency toward saturation, and sufficient sequence depth was set.

### Gene expression analysis

Gene expression in the brain, kidney, and intestine was analyzed using RT-qPCR (Thermal cycler dice real time system, Model TP700/760, Takara, Japan). RNA was isolated from each organ by using a Hybrid-R™ RNA purification kit (GeneAll Biotechnology, South Korea). After the remaining DNA was removed with a Riboclear™ plus kit (GeneAll Biotechnology, South Korea), purity and concentration were evaluated with a NanoDrop Lite spectrophotometer (Thermo Fisher Scientific). cDNA was synthesized with a PrimeScript™ first-strand cDNA synthesis kit (Takara, Japan), and RT-qPCR was performed using primers based on the gene sequence in Table 2 and TB Green™ Premix Ex Taq (Takara, Japan). Relative quantification was calculated by the 2^− ΔΔCT^ method using β-actin as a reference gene.

**Table 2:** Gene specific primers used to quantify relative gene expression.

Gene	Sense	Sequence	Size	Ref
β-actin	F	CATCAGGGAGTGATGGTGGGTA	107	HQ386788.1
	R	ATACCGTGCTCGATGGGGTACT		
TNF-α	F	CAGCAGCGTCACTGCAGAGTTA	120	AB040448.1
	R	GTTACCACCTCACCCCACCATT		
Interferon γ	F	CTGTCTGTCCCTGTGTCTTTGTG	130	AB435094
	R	GGGCTTCCCGTTGAATCTGT		
MX protein	F	GAAAAGAGTTTGGGAAGTGGAACA	174	AB110446
	R	GTAGTTGATGAAGCCTGGCAGTT		
FasL	F	CAGCTGGCTGACCGTGATG	176	AB206382
	R	CTTCTCGTCCCTCGATTTGC		
Caspase3	F	GCAAATCGCTGGTGGGAAA	96	JQ394697
	R	CATCGTCTACACTGTCTGTTTCG		
Caspase10	F	GCACATGGACATCCTGAGTG	201	XM020089584
	R	AGGCTGCTCATTTCACTGCT		
IL-1β	F	CATCACCACTGTCTGCTGGAAA	122	KF025662.1
	R	GCTACTCAACAACGCCACCTTG		
IL-6	F	CAGTGCCAACTTCAGCAAGGAG	130	DQ267937.1
	R	GTGATATCTGGCGTGCAAGAGG		
IL-8	F	CCTCTCTGGCCATCAGTGAAG	194	AF216646
	R	AGTGAGAGTTGGGAGAGATTATTTCC		
IL-10	F	AGCGAACGATGACCTAGACACG	114	KF025662.1
	R	ACCGTGCTCAGGTAGAAGTCCA		
HSP70	F	CAGTCCAGGCTGCTATCCTCAT	111	KY856946.1
	R	TCATGACTCCACCAGCAGTCTC		
HSP90	F	GACTGAACCCACCAAGATGGAC	111	KY856948.1
	R	GCTTTGGTCATACCGATTCCAG		
GH	F	ACCAGAACCAGCCATGAAC	119	M23439.1
	R	CAACAGCGATGGAGAACAG		
GHR	F	TGTCATGGTCAACTGGGAGC	163	AB058418.1
	R	TTTGCCTATGTGCAGACCGT		

### Statistical analysis

Data were statistically analyzed via ANOVA by using Statistical Package for the Social Sciences (SPSS; IBM, USA), followed by Duncan’s multiple range test. Results with *P* < 0.05 were considered significant.

### Ethics approval and consent to participate

This study was conducted under the guidelines of the Animal Ethics Committee Regulation issued by Dong-Eui University (DEU-R2022-031).

## Results

### Bacterial isolation, identification, and characteristics

**Figure 1:**
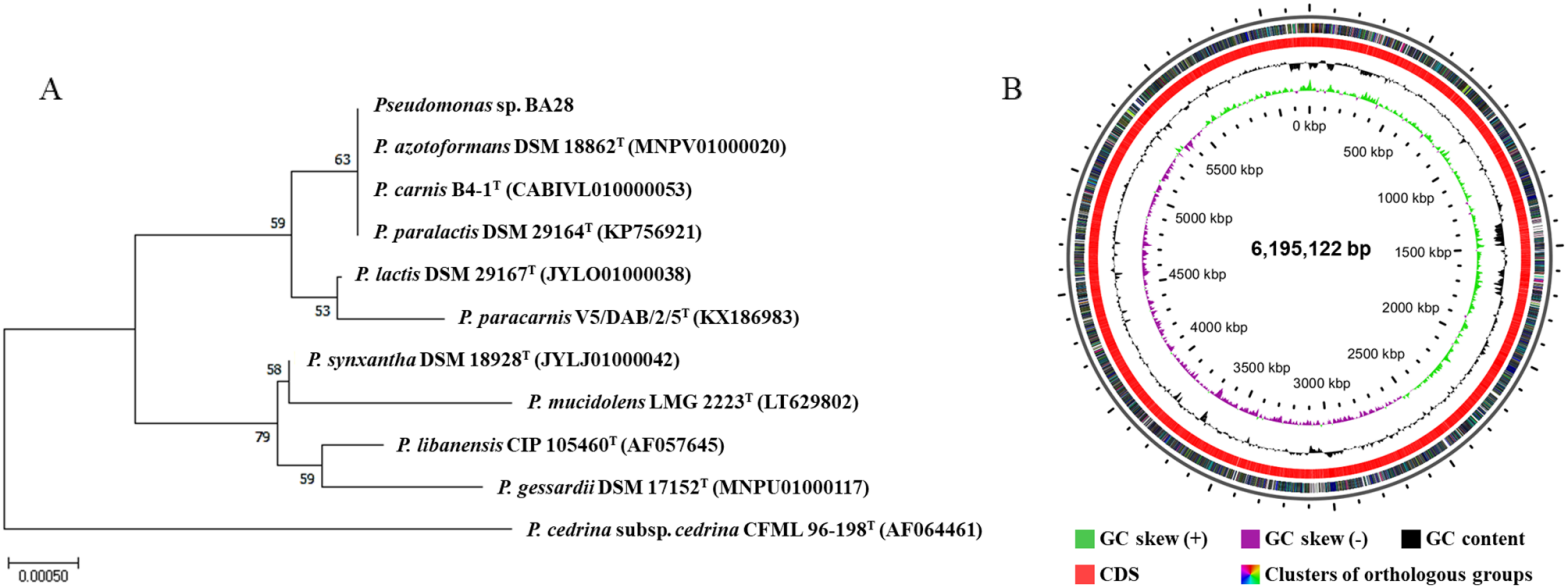
Phylogenetic tree analysis and circular genome visualization of *Pseudomonas* sp. BA28. (**A**) The phylogenetic tree based on 16S rRNA gene sequences was constructed by the neighbor joining method using MEGA 7 with 1000 bootstrap repetitions, after which the 16S rRNA sequences were aligned by the Clustal W program. (**B**) Circles from the center to the outside: the number of bases, GC skew, GC content, location of all annotated ORFs (colored by COG categories).

In bacterial identification based on 16S rRNA sequences, the isolated bacteria shared 100% homology with *Pseudomonas azotoformans* DSM 18862^T^ (MNPV01000020), *P. carnis* B4-1^T^ (MNPV01000020), and *P. paralactis* DSM 29164^T^ (KP756921; Fig. 1A). The isolated strain was named *Pseudomonas* sp. BA28. In general, BA28 can survive at 10 °C–40 °C and pH 4–8. Its optimal growth conditions were identified as 15 °C and pH 6. Its complete genome was circular and had 6,195,122 bp with a 59.9% guanine–cytosine (GC) content. It also had 6,752 protein-encoding genes and 88 RNAs. Figure 1B shows a circular plot of the genome, including the number of bases, GC skew, GC content, and location of all annotated open reading frames sorted by the clusters of orthologous gene (COG) category and colored.

### Growth performance, feed utilization, and body indices

The growth performance and feed utilization parameters of the FM70 and FM35 groups had no significant differences. Conversely, the HALP group supplemented with probiotics showed positive changes compared with those of the two other groups. The final body weight of the HALP group was 37.59 ± 1.29 g, which was significantly increased compared with those of the FM70 (32.21 ± 0.46 g) and FM35 (31.24 ± 0.82 g) groups. Variations in the final body weight caused significant differences in the weight gain and specific growth rate among the three groups. The feed conversion ratio of the HALP group (1.06 ± 0.07) was significantly decreased compared with those of the FM70 (1.25 ± 0.03) and FM35 (1.26 ± 0.06) groups. The protein efficiency ratio of the HALP group (1.85 ± 0.13) was significantly increased compared with those of the FM70 (1.57 ± 0.04) and FM35 (1.55 ± 0.10) groups. The condition factor and organosomatic indices did not significantly differ among the three groups (Table 3).

**Table 3:** Growth performance, feed utilization and organosomatic indices of olive flounder fed the experimental diets.

Groups	Growth performance, feed utilization and organosomatic parameters							
	FBW (g)	WG (%)	SGR (% day^−1^)	FCR	PER	CF (%)	VSI (%)	HSI (%)
FM70	31.21 ± 0.46^a^	190.33 ± 4.28^a^	1.90 ± 0.03^a^	1.25 ± 0.03^b^	1.57 ± 0.04^a^	0.90 ± 0.04	2.88 ± 0.04	1.61 ± 0.13
FM35	31.24 ± 0.82^a^	187.96 ± 7.58^a^	1.89 ± 0.05^a^	1.26 ± 0.06^b^	1.55 ± 0.10^a^	0.88 ± 0.03	2.95 ± 0.06	1.51 ± 0.06
HALP	37.59 ± 1.29^b^	245.81 ± 11.84^b^	2.21 ± 0.06^b^	1.06 ± 0.07^a^	1.85 ± 0.13^b^	0.92 ± 0.06	2.94 ± 0.14	1.61 ± 0.16

*WG* weight gain = 100 × (Final weight − Initial weight)/Initial weight, *SGR* specific growth rate = 100 × (ln final weight − ln initial weight)/days, *FCR* feed conversion ratio = Dry feed intake/Wet body WG, *PER* protein efficiency ratio = Wet body WG/Protein fed, *CF* condition factor = 100 × Body weight/(Total body length)3, *VSI* viscerosomatic index = 100 × (Visceral weight/Body weight), *HSI* hepatosomatic index = 100 × (Liver weight/Body weight), Values are mean ± SD of three replicates. Values with different superscript letters within the same column in the table are significantly different (P < 0.05). The lack of superscript letter indicates no significant differences (P > 0.05). FM70, basal diet; FM35, low-fishmeal diet; HALP, low-fishmeal diet supplemented with host-associated low-temperature probiotics.

### Serum nonspecific immune and biochemical parameter analysis

**Figure 2:**
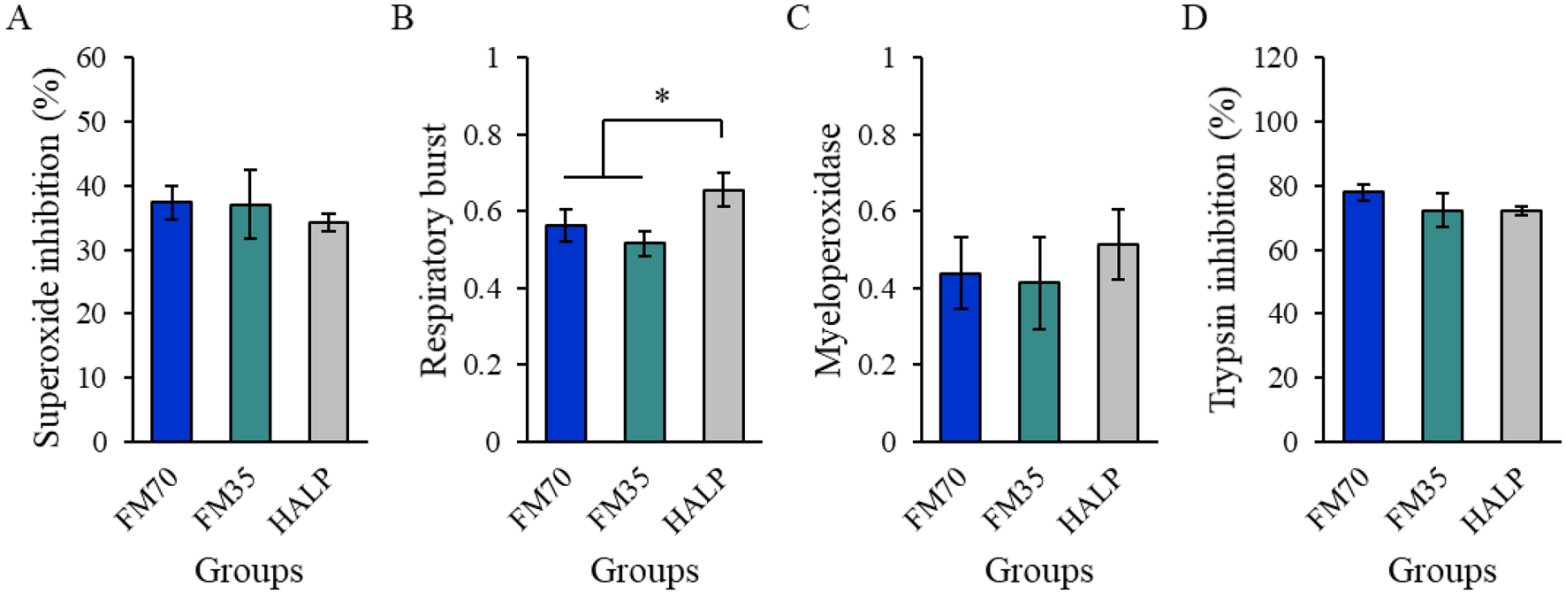
Serum non-specific immune parameters ((**A**) superoxide dismutase activity; (**B**) Respiratory burst activity; (**C**) myeloperoxidase activity; (**D**) antiprotease activity) of olive flounder fed the experimental diets. Data represent the mean ± standard deviation of 3 replicates (3 fish/replicate); ns means no significant difference (*P* > 0.05) and star means significant difference (*P* < 0.05). *FM70* basal diet, *FM35* low-fishmeal diet, *HALP* low-fishmeal diet supplemented with host-associated low-temperature probiotics.

**Figure 3:**
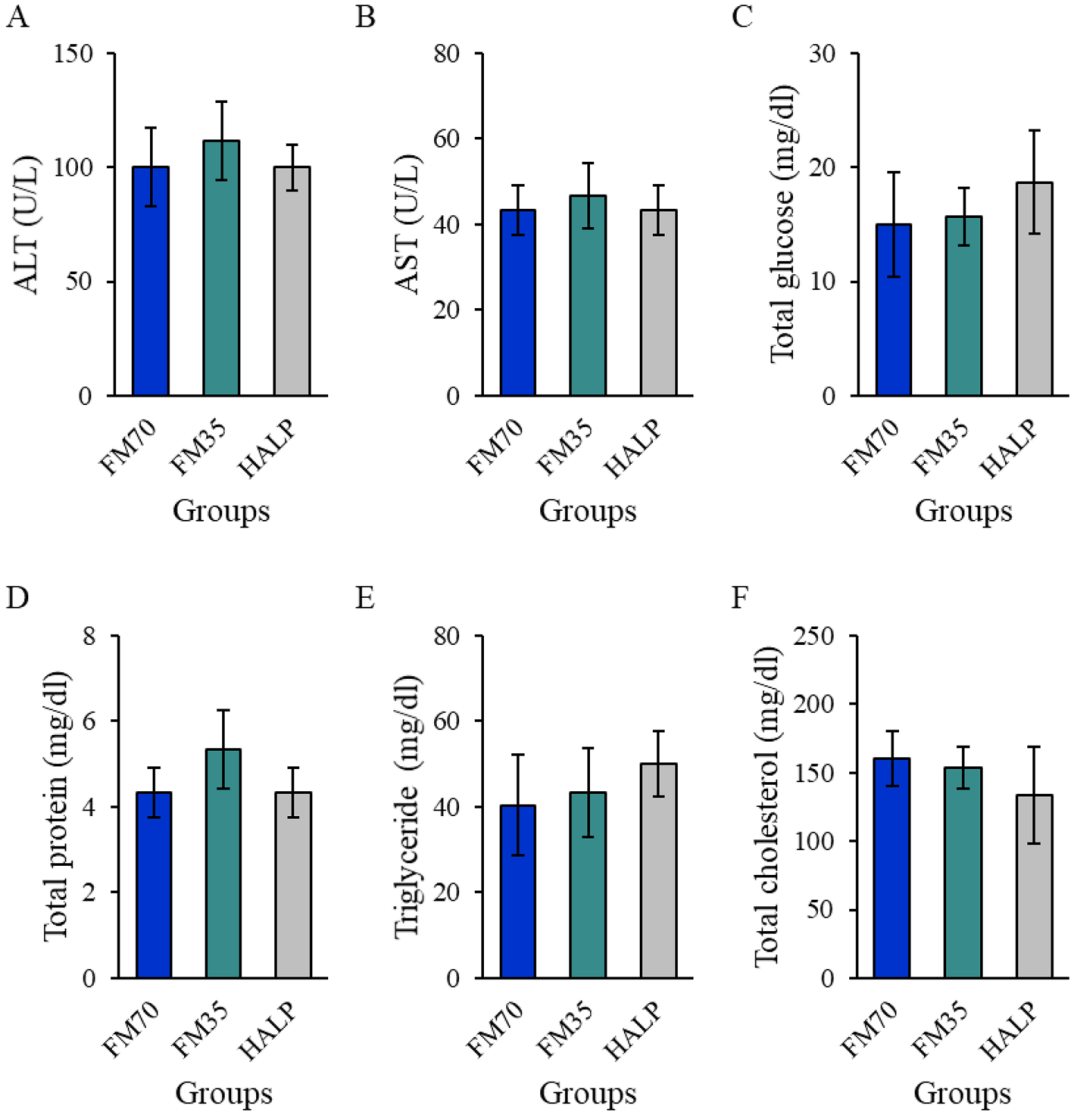
Biochemical parameters ((**A**), alanine aminotransferase; (**B**), aspartate aminotransferase; (**C**), total glucose; (**D**), total protein; (**E**), triglyceride; (**F**), total cholesterol;) of olive flounder fed the experimental diets. Data represent the mean ± standard deviation of 3 replicates (3 fish/replicate); ns means no significant difference (*P* > 0.05) and star means significant difference (*P* < 0.05). *FM70* basal diet, *FM35* low-fishmeal diet, *HALP* low-fishmeal diet supplemented with host-associated low-temperature probiotics.

The nonspecific immune parameters in the respiratory burst showed significant differences. Such parameters in the HALP group (0.66 ± 0.05) increased compared with those in the FM70 (0.56 ± 0.04) and FM35 (0.52 ± 0.03) groups. Other parameters (superoxide dismutase, myeloperoxidase, and antiprotease activity; Fig. 2) did not significantly differ among the three groups. All biochemical parameters did not also significantly vary among the three groups (Fig. 3).

### Digestive enzyme activity

**Figure 4:**
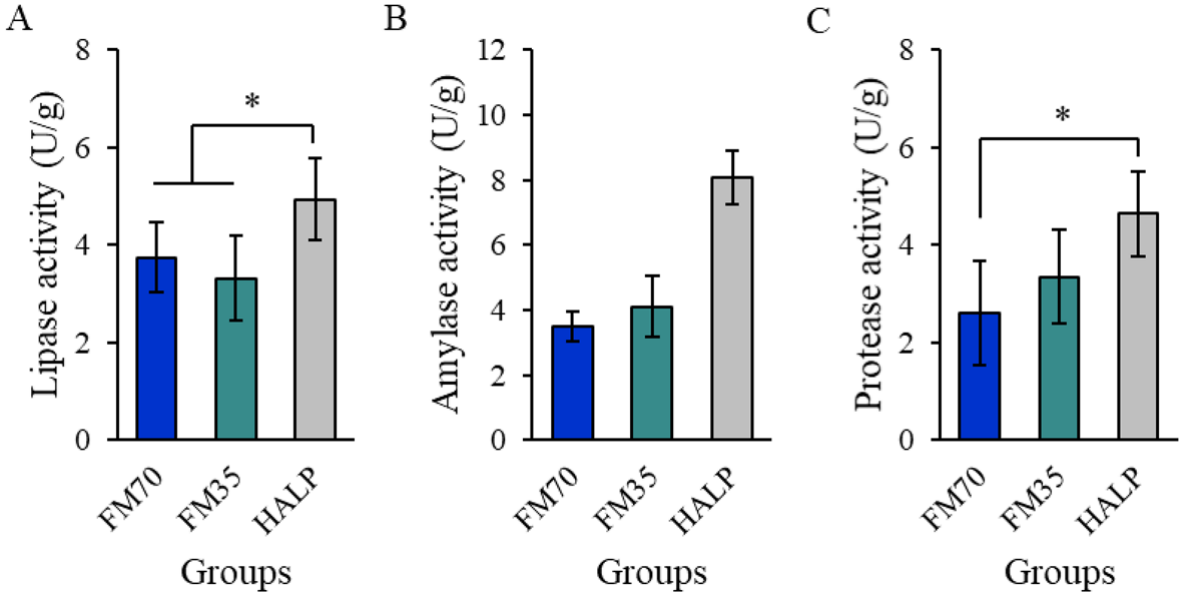
Amylase (**A**), lipase (**B**), and protease (**C**) activities in intestine after 8-month feeding trial in olive flounder. Data represent the mean ± standard deviation; means that do not share the same letter differ significantly (*P* < 0.05). *FM70* basal diet, *FM35* low-fishmeal diet, *HALP* low-fishmeal diet supplemented with host-associated low-temperature probiotics.

Digestive enzyme activities, specifically amylase and protease activities, in the intestine showed significant differences. The amylase activity significantly increased in the HALP group (8.08 ± 0.84 U/g) compared with that in the FM70 (3.49 ± 0.45 U/g) and FM35 (4.11 ± 0.94 U/g) groups. The protease activity significantly differed only between the HALP (4.64 ± 0.88 U/g) and FM70 (2.61 ± 1.08 U/g) groups. The lipase activity did not significantly differ among the three groups (Fig. 4).

### Intestinal microbiota analysis

**Figure 5:**
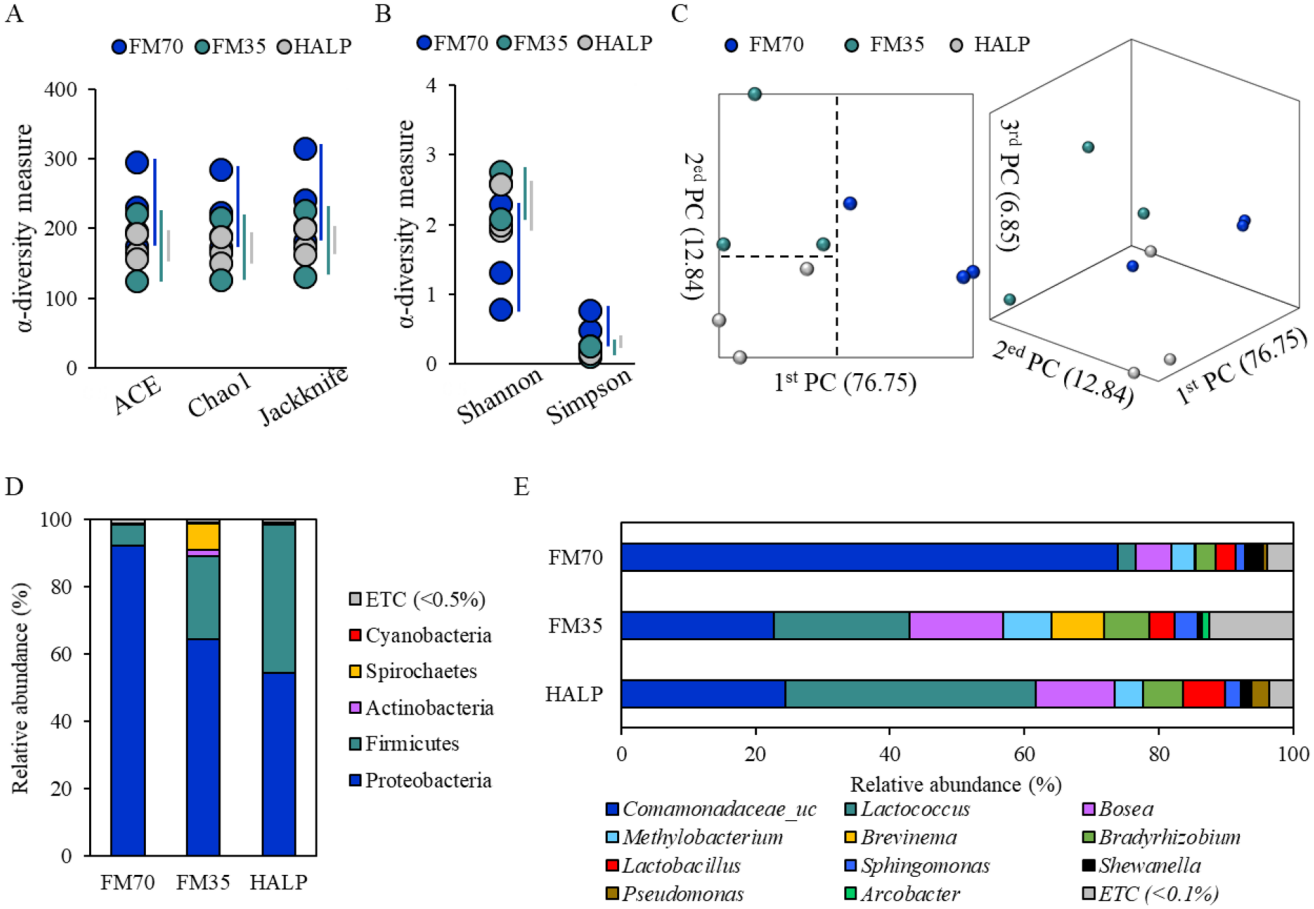
Intestinal microbiota analysis of olive flounder fed experimental diet. α-diversity of the bacterial communities (**A**,**B**). β-diversity based on principal coordinates analysis (**C**). Composition and relative abundance of intestinal bacterial communities at the phylum (**D**) and genus (**E**) level. ns means no significant difference (P > 0.05). *FM70* basal diet, *FM35* low-fishmeal diet, *HALP* low-fishmeal diet supplemented with host-associated low-temperature probiotics.

All α-diversity estimates (ACE, Chao1, Jackknife, Shannon, and Simpson) did not significantly differ among the three groups (Fig. 5A,B). β-diversity analysis based on the UniFrac metric via principal coordinate analysis revealed clear boundaries between the three groups. The FM70 group was separated from the two other groups in the first PC, and the separation from the FM35 and HALP groups was confirmed in the second PC (Fig. 5C). The difference between the three groups in the composition of bacteria at the phylum level is the relative abundance of Proteobacteria and Firmicutes. Proteobacteria and Firmicutes accounted for 92.41% and 6.06% in the FM70 group, 64.44% and 24.72% in the FM35 group, and 54.50% and 44.05% in the HALP group, respectively (Fig. 5D). At the genus level, 73.90% of the total bacterial communities in the FM70 group were *Comamonadaceae_uc* and 22.72% and 24.52% in the FM35 and HALP groups, respectively. The relative abundance of *Lactococcus* and *Lactobacillus* was higher in the FM35 (20.24% and 3.80%) and HALP (37.14% and 6.16%) groups than in the FM70 (2.59% and 3.06%) group (Fig. 5E).

### Gene expression analysis

**Figure 6:**
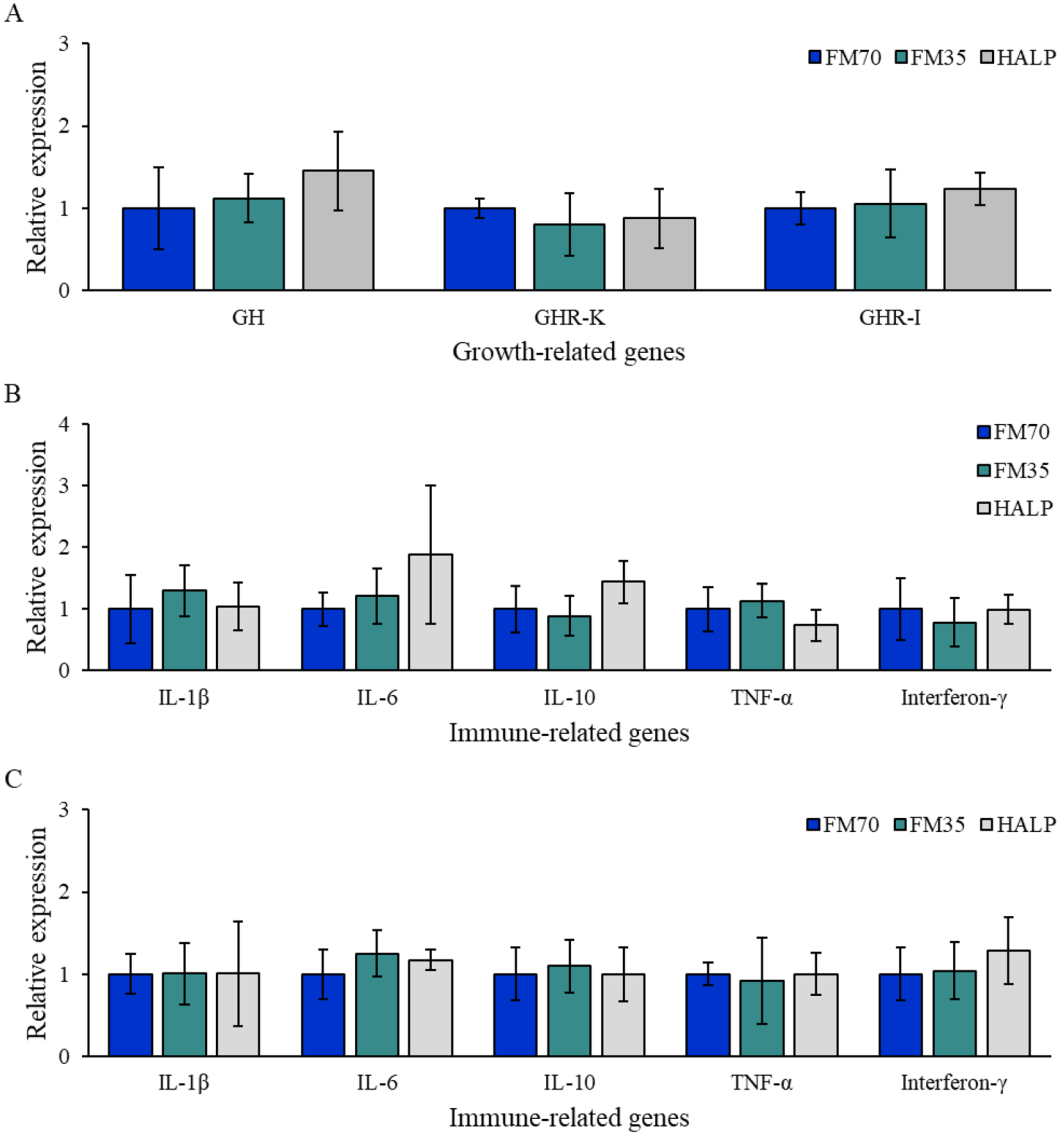
Profiles of growth (**A**) and immune (**B**,**C**) related genes expression of olive flounder as measured by RT-qPCR. The gene expression level of growth hormone (GH) was investigated in the brain, and the gene expression of growth hormone receptor (GHR) was investigated in the kidney (GHT-K) and intestine (GHT-I). Immune-related gene expression was investigated in both kidney (**B**) and intestine (**C**). Gene expression was quantified relative to β-actin transcription. Data represent the mean ± standard deviation of 3 replicates (3 fish/replicate); ns means no significant difference (P > 0.05) and star means significant difference (P < 0.05). *FM70* basal diet, *FM35* low-fishmeal diet, *HALP* low-fishmeal diet supplemented with host-associated low-temperature probiotics, *IL* interleukin, *TNF* tumor necrosis factor.

**Figure 7:**
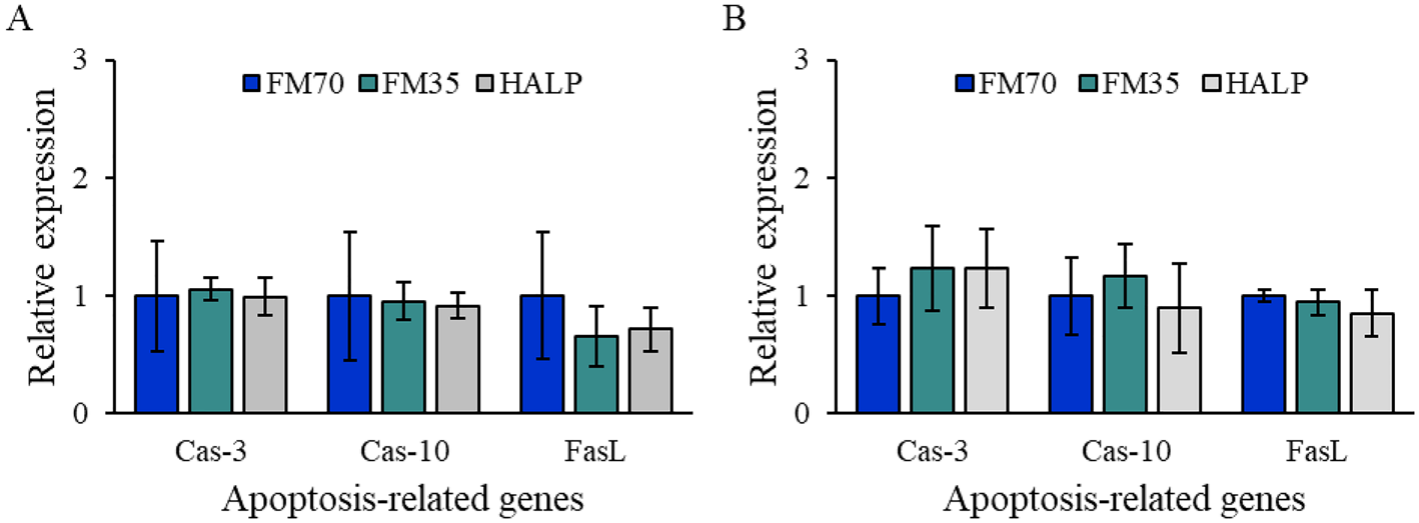
Profiles of apoptosis related genes expression of olive flounder as measured by RT-qPCR. The gene expression level was investigated in the kidney (**A**) and intestine (**B**). Gene expression was quantified relative to β-actin transcription. Data represent the mean ± standard deviation of 3 replicates (3 fish/replicate); ns means no significant difference (P > 0.05). *FM70* basal diet, *FM35* low-fishmeal diet, *HALP* low-fishmeal diet supplemented with host-associated low-temperature probiotics, *Cas* caspase, *FasL* FAS ligand.

RT-qPCR was performed to compare the expression levels of growth- and immune-related genes in olive flounder fed with each experimental diet. The gene expression of growth hormone in the brain and the growth hormone receptor in the kidney and intestine did not significantly differ in all groups (Fig. 6A). Immune-related gene expression levels between the three groups were compared in the kidney and intestine. The expression levels of the five genes did not significantly vary among the three groups (Fig. 6B,C). The expression levels of the apoptosis-related genes in the kidney and intestine did not significantly differ among the three groups (Fig. 7).

## Discussion

In this study, HAPs were isolated from the intestine of healthy wild olive flounder, and the isolated strain was identified as a *Pseudomonas* species through whole genome sequencing. Various probiotic strains for farmed fish have been studied, but studies on HAPs are rare. They can adapt to the intestinal environment of the host better than candidate strains from other sources and cause various effects, such as increasing the rate of nutrient digestion and absorption by producing enzymes and metabolites that show high activity in the host’s intestinal environment^17,21,24^. In the present study, low-temperature probiotics showing the optimal growth rate at 15–20 °C were selected as candidate strains. Most probiotics, including *Bacillus* species and lactic acid bacteria, developed for farmed olive flounder are strains that show high growth rates at approximately 37 °C^3,10,11,19,20^. However, evidence is insufficient to show whether these strains as probiotics provide beneficial effects to the host at 15–20 °C, which is the growth temperature of olive flounder. In this context, the host-associated low-temperature probiotics proposed in this study for the first time would be suitable for the development of probiotics for farmed fish in terms of their ability to colonize in the host’s intestine and provide beneficial effects.

Probiotics studied in farmed fish provide many positive effects, including improved growth performance^19^. Probiotics not only improve the appetite of farmed fish but also increase their digestibility by breaking down indigestible substances such as phytic acid^25^. Consequently, their growth increases, and feed utilization improves. Liu et al.^26^ demonstrated that *Bacillus* species produce various enzymes that efficiently metabolize carbohydrates, lipids, and proteins, and their activities help improve the growth performance of the host. In the present study, BA28 was confirmed to have various enzyme genes related to carbohydrate, protein, and lipid metabolism through WGS analysis. In addition, microorganisms such as *Pseudomonas* species that survive at relatively low temperatures are known to produce enzymes that show higher activity at low temperatures than those produced by *Bacillus* species, which have an optimum temperature of 37 °C. In fact, phytase produced by *Bacillus* species shows a high activity at 50 °C or higher, whereas phytase produced by *Pseudomonas* species exhibits a high activity at 25–40 °C^27^. This finding indicates that enzymes produced by *Pseudomonas* species that survive at low temperatures are more appropriate than those produced by *Bacillus* species to increase digestibility by decomposing phytic acid, an indigestible substance in the intestine of olive flounder living at approximately 20 °C^27^. These results suggest that probiotics with high growth at a temperature similar to the growth temperature of the host have more potential to provide beneficial effects; in the case of olive flounder, low-temperature probiotics with high growth at 15–20 °C are more effective than high-temperature probiotics.

Among the many beneficial effects of probiotics, immune system modulation is one of the most recognized benefits^28^. Immunological studies on several probiotic-fed fish have shown that several probiotics, individually or in combination, can improve fish immunity^28^. Conversely, the provision of probiotics in this study did not cause significant differences in SOD, MPO, and antiprotease activity among the innate immunity parameters of flounder. The probiotics used in this study were not newly introduced strains in the intestines of flounder, but they are existing microorganisms isolated from the intestines of flounder; therefore, they may not affect the regulation of innate immunity. However, few studies have been conducted on the effects of HAP in fish. Therefore, more research is needed to fully understand the effects of HAPs on the regulation of innate immunity in fish.

Probiotics produce various enzymes that aid the host’s digestion; when applied appropriately to fish, they not only increase feed efficiency and growth but also eliminate antinutritional factors present in the feed and prevent intestinal disorders and pre-digestion^29^. Sankar et al.^30^ reported that microbial-derived exogenous enzymes have a wide pH range, are active throughout digestion, and may allow for better substrate hydrolysis. Thus, they suggest supplementing the diet with probiotics may improve digestive enzyme activities^30^. As described above, the probiotics used in this study were derived from the host’s intestine, suggesting that they adapt well to the host’s intestinal environment. Moreover, the enzymes they produce likely remain highly active in the host’s intestinal environment. As a result, these properties can increase the digestibility of olive flounder by increasing digestive enzyme activities.

The gut microbiome influences the host’s gut development, immunity, and metabolism, and an imbalanced fish gut microbiome causes decreased metabolism, growth, stress, and disease development^31^. The gut microbiome is primarily formed by diet and can be modified by antibiotics, immunostimulants, prebiotics, and probiotics^32,33^. Among the changes induced by HALP supplementation in this study, a notable part was the increase in the relative abundance of lactic acid bacteria such as *Lactobacillus* and *Lactococcus*. Lactic acid bacteria are generally recognized as beneficial microorganisms because of their ability to stimulate the host’s gastrointestinal tract development, digestive function, mucosal tolerance, and disease resistance^34^. *Lactobacillus*, *Lactococcus*, *Leuconostoc*, *Enterococcus*, *Streptococcus*, *Carnobacterium*, *Weissella*, and *Pediococcus* exist as indigenous species in the intestine of fish, and they have attracted attention as probiotics for aquaculture because they can produce antibacterial substances against potential fish pathogens. Therefore, the HALP dietary supplementation can provide positive effects by increasing the beneficial bacteria in the intestine of flounder, and the increase in growth performance and digestive enzyme activities may be the result of changes in the intestinal microbiota. Although *Pseudomonas* species used as HALPs in this study were confirmed to increase the abundance of LAB by causing changes in the composition of the intestinal microflora of flounder, the exact mechanism could not be determined from the results of this study. Previous studies have reported that bacteriocins, such as pediocins, produced by LAB can exert antagonistic effects on a variety of bacteria, including gram-negative and gram-positive species, thereby increasing their relative abundance^35–37^. Nevertheless, because the strain used in this study is not a LAB, further research is needed to determine the mechanism of these changes.

Growth hormone and growth hormone receptor play an important role in fish growth and can be used as good biomarkers for estimating growth rates in various fish^38,39^. Previous studies have reported that probiotic supplementation can affect growth and immune-related gene expression in fish^40,41^. In the present study, the comparison of growth-related gene expression revealed no significant difference in any group, confirming no genetically related differences in growth and proving that the increase in growth by HALPs was not associated with the gene expression level. These results indicate that the increase in the growth performance of fish in this study was independent of gene expression and may be related to nutrient digestion and absorption. This could have the advantage of being safe to use, as there are no other physiological effects that may occur due to changes in gene expression. Similarly, a study using a cell-based laboratory model of the salmon gut reported that beneficial microbes had no effect on the expression of key intestinal barrier and immune molecules^42^.

Various probiotics affect the expression of immune-related genes in fish^19^. Previous reports showed that the moderate transcription of immune-related genes, such as pro-inflammatory cytokines, helps maintain immune balance and increase resistance to infection^19,43^. Peptidoglycan, lipopolysaccharide, flagella, and nucleic acids, which are constituents of various probiotics, are known as microbe-associated molecular patterns (MAMPs) that induce the transcription of pro-inflammatory cytokines^19,44^. The binding of MAMPs to pathogen pattern recognition receptors on dendritic cells or Toll-like receptors activates and stimulates macrophages and T cells to induce the transcription of pro-inflammatory cytokines^45–47^. However, similar to the innate immune response in this study, immune-related gene expression was not significantly affected by the dietary supplementation of HALPs. Since HALPs were isolated from the gut of the host, they did not appear to stimulate the host’s immune-related response. Similarly, HALPs did not significantly affect apoptosis-related gene expression, indicating that they did not exert any toxic effect on host cells.

## Conclusion

The HALPs isolated in this study improved the growth performance of olive flounder by positively changing the digestive activity and intestinal microbial composition without affecting host gene expression. They could be used as novel feed additives for olive flounder farming.

## Data Availability

The datasets used and/or analysed during the current study are available from the corresponding author on reasonable request.

## Abbreviations

ALT: Alanine aminotransferase
AST: Aspartate aminotransferase
BHI: Brain heart infusion
CF: Condition factor
COG: Clusters of orthologous gene
FCR: Feed conversion ratio
GC: Guanine–cytosine
HALPs: Low-temperature probiotics
HAPs: Host-associated probiotics
HIS: Hepatosomatic index
MAMPs: Microbe-associated molecular patterns
MPO: Myeloperoxidase
PBS: Phosphate-buffered saline
PER: Protein efficiency ratio
RB: Respiratory burst
SGR: Specific growth rate
SOD: Superoxide dismutase
VSI: Viscerosomatic index
WG: Weight gain
WGS: Whole-genome sequence
